# MiR-143 acts as a tumor suppressor by targeting N-RAS and enhances temozolomide-induced apoptosis in glioma

**DOI:** 10.18632/oncotarget.2116

**Published:** 2014-06-18

**Authors:** Lin Wang, Zhu-mei Shi, Cheng-fei Jiang, Xue Liu, Qiu-dan Chen, Xu Qian, Dong-mei Li, Xin Ge, Xie-feng Wang, Ling-Zhi Liu, Yong-ping You, Ning Liu, Bing-Hua Jiang

**Affiliations:** ^1^ State Key Lab of Reproductive Medicine, Department of Pathology, Nanjing Medical University, Nanjing, China; ^2^ Department of Neurosurgery, The First Affiliated Hospital of Nanjing Medical University, Nanjing, China; ^3^ Department of Pathology, Anatomy and Cell Biology, Thomas Jefferson University, Philadelphia, USA

**Keywords:** miR-143, N-RAS, tumor growth, Glioma

## Abstract

Therapeutic applications of microRNAs (miRNAs) in RAS-driven glioma were valuable, but their specific roles and functions have yet to be fully elucidated. Here, we firstly report that miR-143 directly targets the neuroblastoma RAS viral oncogene homolog (N-RAS) and functions as a tumor-suppressor in glioma. Overexpression of miR-143 decreased the expression of N-RAS, inhibited PI3K/AKT, MAPK/ERK signaling, and attenuated the accumulation of p65 in nucleus of glioma cells. In human clinical specimens, miR-143 was downregulated where an adverse with N-RAS expression was observed. Furthermore, overexpression of miR-143 decreased glioma cell migration, invasion, tube formation and slowed tumor growth and angiogenesis in a manner associated with N-RAS downregulation *in vitro* and *in vivo*. Finally, miR-143 also sensitizes glioma cells to temozolomide (TMZ),the first-line drug for glioma treatment. Taken together, for the first time, our results demonstrate that miR-143 plays a significant role in inactivating the RAS signaling pathway through the inhibition of N-RAS, which may provide a novel therapeutic strategy for treatment of glioma and other RAS-driven cancers.

## INTRODUCTION

Glioma, the most common and aggressive form of primary brain tumor, presents a dismal prognosis. The poor prognosis and high lethality of the disease is largely due to the highly invasive/migratory nature of glioma cells, which are capable of diffusely infiltrating and widely migrating in the surrounding brain tissue [1-7]. Despite treating with combinations of surgery, radiotherapy and chemotherapy, glioblastomamultiforme (GBM), the most malignant and most common glioma, is associated with an average life expectancy as short as only 15 months [8]. Temozolomide (TMZ), a DNA alkylating antineoplastic drug, has been used universally in glioma treatment by inhibiting the proliferation of glioma cells and inducing apoptosis [9]. However, the patients′response rate to TMZ-based chemotherapy still remains modest, mainly due to the development of drug resistance. Thus, it is essential to developing novel and effective therapeutic strategies for glioma.

MicroRNAs are small noncoding regulatory RNA molecules, with profound impact on a wide array of biological processes [10, 11]. MicroRNAs have been recently implicated in the regulation of tumorigenesis, differentiation, proliferation, and survival through the inhibition of major cellular pathways [12, 13]. Among them, miR-143 has been demonstrated to function as a tumor suppressor, and loss of miR-143 expression has been reported in many cancer types, restoration of miR-143 expression has been shown to abrogate tumorigenesis [14]. To date, some genes have been identified as miR-143 target genes, including K-RAS, ELK1, MYO6, Bcl-2, ERK5 and IGF-IR, that are involved in pathogenesis of cancers [15-21]. However, the molecular mechanism of miR-143 repression in glioma has not been determined.

RAS sarcoma (Ras) genes are the most frequently activated oncogenes in human cancer. K-RAS, N-RAS and H-RAS represent the prototype members of a family of small G proteins that are frequently activated to an oncogenic state in a wide variety of human tumors, including glioma. K-RAS has been reported to be a target of miR-143 in colorectal cancer cell. N-RAS, as one of the three members of the RAS oncogene family, encode small GTPases that are involved in cellular signal transduction. N-RAS is implicated and functionally altered in a number of cancers including glioma and has potential roles in the regulation of cancer cell growth, survival, migration, invasion, and angiogenesis [22, 23]. Enhanced expression of N-RAS gene by disorder of transcription may be a factor in the progression of glioma [24]. Oncogenic N-RAS promotes tumorigenesis through activation of multiple downstream pathways, including PI3K/AKT, MAPK/ERK, and NF-κB pathway [25-28]. Thus, N-RAS silencing has become an efficient therapeutic strategy in glioma, but it is still far from optimal and novel therapeutic strategies are needed urgently.

In the present study, we demonstrated that miR-143 was downregulated in human glioma. Then, we will ask several important questions in this study: (1) what are the roles of miR-143 in tumor growth and angiogenesis; (2) what is the potential direct target of miR-143 that may be associated with cancer development; and (3) whether miR-143 overexpression inhibits AKT, ERK1/2, and NF-κB signaling pathways via its direct target; (4) What role of miR-143 and underlying mechanisms in glioma resistance to TMZ treatment. The answers of these questions would provide new insights into the molecular mechanism of glioma development as well as provide new therapeutic strategy for glioma treatment in the future.

## RESULTS

### MiR-143 expression was downregulated in glioma

**Figure1:**
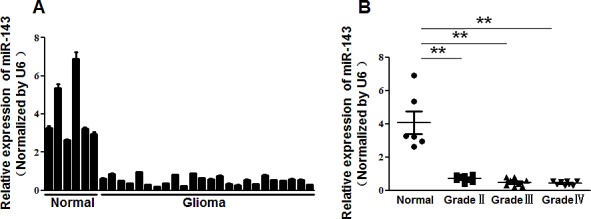
MiR-143 expression was downregulated in glioma (A)Relative miR-143 expression levels were analyzed by qRT-PCR in 6 normal brain tissues and 24 glioma tissues. U6 RNA level was used as an internal control. (B) The levels of miR-143 expression in normal brain tissues and three different grades of glioma samples. According to the pathological classification, the 24 glioma tissues were divided into three groups (8 glioma tissues each group): WHO Grade II, Grade III and Grade IV. Data represent mean±SD of 3 replicates. ** indicated significant difference at *P*<0.01.

To investigate the role of miR-143 in glioma, we first evaluated the expression levels of miR-143 in normal brain tissues and glioma tissues by qRT-PCR (Figure 1A). The results showed that the expression levels of miR-143 were consistently lower in the glioma tissues than in normal brain tissues. By dividing all glioma samples into Grade II, Grade III, and Grade IV according to WHO classification, we found that miR-143 levels were downregulated in these 3 groups compared to the normal brain group. But the levels of miR-143 expression among high grade tumors (WHO Grades III and IV) showed no significantly difference to those in low grade tumors (WHO Grade II) (Figure 1B). Our results indicated that the loss of miR-143 might be an early event in gliomagenesis.

### N-RAS is a direct target of miR-143

**Figure2:**
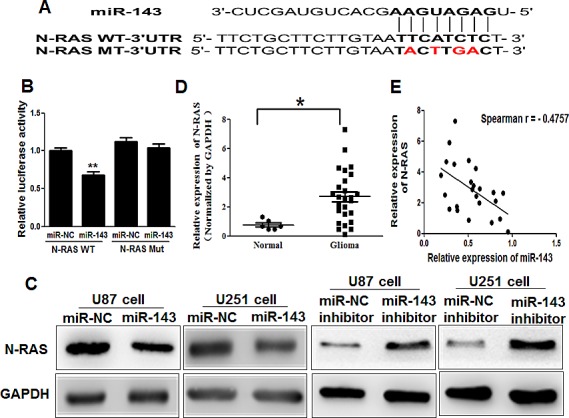
N-RAS is a direct target of miR-143 (A)Sequence of the miR-143-binding site within the human N-RAS 3′-UTR and a schematic diagram of the reporter construct showing the entire N-RAS 3′-UTR sequence and the mutated N-RAS 3′-UTR sequence. The mutated nucleotides of the N-RAS 3′-UTR are labeled in red. (B)Luciferase assay on U87 cells, which were co-transfected with miR-NC or miR-143 and a luciferase reporter containing the full length of N-RAS 3′-UTR (WT) or a mutant(Mut) in which four nucleotides of the miR-143-binding site were mutated. Activities were measured 24 hours post transfection. MiR-143 markedly suppressed luciferase activity in N-RAS3′-UTR (WT) reporter constructs. The data are means ± SEM for separate transfections (n=4). (C)The immunoblotting showed that expression levels of N-RAS were decreased in cells with miR-143 overexpression, but increased in cells with anti-miR-143 inhibitor. (D)The expression levels of N-RAS in normal human brain tissues and glioma specimens were determined by Western blotting analysis, and fold changes were obtained from the ratio of N-RAS to GAPDH levels. (E)Spearman's correlation analysis was used to determine the correlation between the expression levels of N-RAS and miR-143 in human glioma specimens. Data represent mean±SD of 3 replicates. *, ** indicated significantly different at *P*<0.05, *P<*0.01, respectively.

To fully understand the mechanisms of miR-143 in glioma, TargetScan search program was used to predict targets of miR-143, which N-RAS has been thought to be putative target of miR-143 (Figure 2A). U87 cells were cotransfected with the wild (WT) or mutated (Mut) N-RAS luciferase reporter vector together with miR-143 or miR-NC for 24 h, and luciferase activities in those cells were measured. As shown in Figure 2B, luciferase activities were significantly reduced in those cells transfected with the wild sequence and miR-143, but not in the cells with the mutant sequence and miR-143. Then, Western blotting analysis was conducted to measure the levels of N-RAS protein, we found that the expression of N-RAS protein was downregulated in miR-143 treated cells, but increased in cells transfected with anti-miR-143 inhibitor (Figure 2C). These results suggest that miR-143 directly targets N-RAS by binding its seed region to their 3'-UTRs in glioma cells.

Furthermore, we measured the levels of N-RAS protein in glioma specimens and normal brain tissues. The results showed that the average expression levels of N-RAS were significantly higher in tumor tissues than those in the normal brain tissues (Figure 2D). Then, we determine the correlation between N-RAS levels and miR-143 expression levels in the same glioma tissues. As shown in Figure 2E, Spearman's rank correlation analysis showed that the expression levels of N-RAS and miR-143 in 24 glioma specimens were inversely correlated (Spearman's correlation r=−0.4757).

### MiR-143 inhibits AKT and ERK1/2 pathways as well as the expression of HIF-1α and VEGF via targeting N-RAS

**Figure3:**
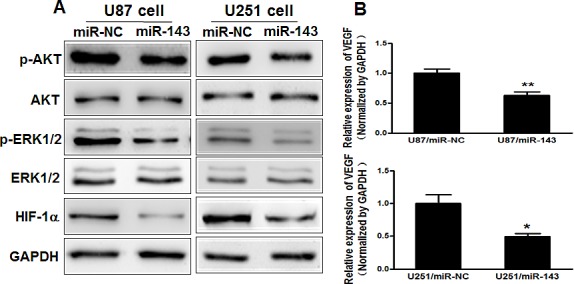

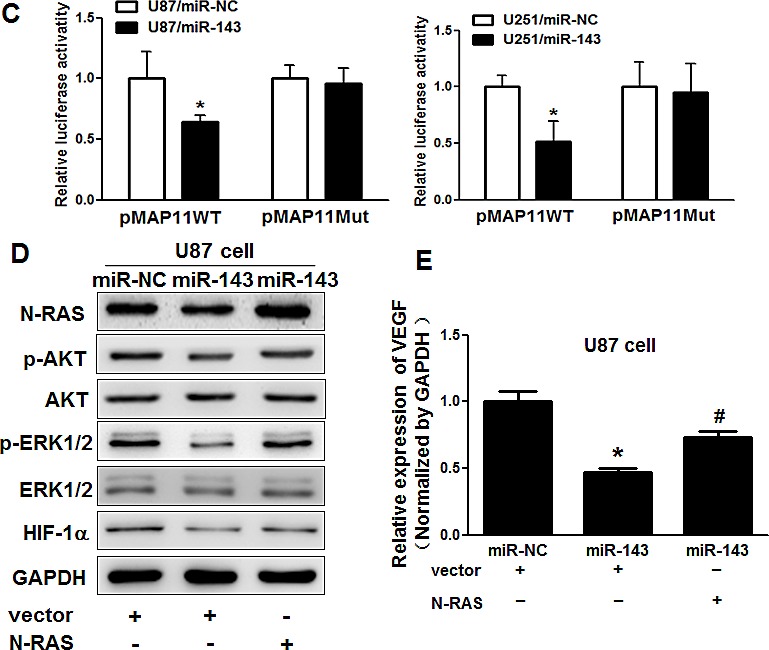
MiR-143 inhibits AKT and ERK1/2 pathways as well as the expression of HIF-1α and VEGF via targeting N-RAS (A) The expression levels of phosphorylated AKT (p-AKT), phosphorylated ERK1/2 (p-ERK1/2) and HIF-1α were decreased in cells with miR-143 overexpression detected by immunoblotting, while AKT and ERK1/2 protein levels were not changed. (B) VEGF levels were measured by qRT-PCR in miR-NC and miR-143 stable cell lines and normalized to level of GAPDH. (C) VEGF reporter plasmid pMAP11-WT or pMAP11-mut was cotransfected with pRL-TK plasmid and equal amounts of miR-NC, miR-143. Firefly and Renilla luciferase activities were measured 24 h later. (D) Overexpression of N-RAS restored miR-143-inhibited levels of p-AKT,p-ERK1/2 and HIF-1α. (E) Overexpression of N-RAS rescued VEGF mRNA expression inhibited by miR-143. The VEGF mRNA levels were normalized to that of GAPDH. Data represent mean±SD of 3 replicates. *indicates significant difference compared to control, ^#^indicates significant difference compared to miR-143 treatment alone at *P*<0.05.

The AKT and ERK1/2 pathways act as major downstream of RAS signaling, and several downstream factors such as hypoxia-inducible factor 1α (HIF-1α) and vascular endothelial growth factor (VEGF) have been linked to the AKT and ERK1/2 pathway. Cellular levels of p-AKT and p-ERK1/2 were significantly decreased in U87 and U251 cells stably expressing miR-143 compared with miR-NC, while no statistically significant reduction of AKT and ERK1/2 was detected (Figure 3A).

Previous studies have shown the importance of HIF-1α and VEGF in the regulation of angiogenesis in glioma [29, 30]. Here, we observed that HIF-1α and VEGF levels in miR-143-expressing U87 and U251 cells were both downregulated (Figure 3A and B). It has been reported that HIF-1α activates the expression of VEGF gene by binding to the hypoxia response element (HRE) in the VEGF promoter region. To determine whether miR-143 inhibits VEGF expression through HIF-1α DNA binding site of VEGF promoter, we analyzed the effects of miR-143 on a pMAP11WT VEGF promoter reporter plasmid containing the HIF-1α binding site, and a pMAP11Mut plasmid which 3 bp substitution at the HIF-1α binding site. As shown in Figure 3C, miR-143 inhibited the pMAP11WT reporter activity in U87 and U251 cells, but not pMAP11Mut reporter activity, which indicating that miR-143 inhibits the expression of VEGF through HIF-1α. To further explore whether the overexpression of N-RAS influence the expression of p-AKT, p-ERK1/2, HIF-1α and VEGF, U87 cells were cotransfected with or without pCMV6-N-RAS cDNA, these data provide evidence that miR-143 inhibits AKT and ERK1/2 pathways as well as the expression of HIF-1α and VEGF via targeting N-RAS (Figure 3D and E).

### MiR-143 attenuates the accumulation of p65 in nuclear in glioma cells

**Figure4:**
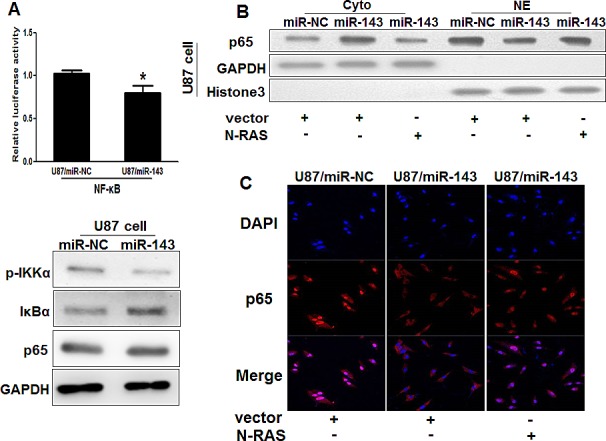
MiR-143 attenuates the accumulation of p65 in nucleus in glioma cells (A) Luciferase assay of the interaction between NF-κB signaling and miR-143 in U87 cells. Immunoblotting analysis of IKKα, IκBα, p65 and GAPDH in U87/miR-NC and U87/miR-143 cells. (B)Immunoblotting analysis of p65, GAPDH in cytoplasmic extracts (Cyto) and p65, Histone 3 in nuclear extracts (NE) in U87/miR-NC,U87/miR-143,and U87/miR-143 overexpressed with N-RAS. (C) Immunofluoresence assay was performed on U87/miR-NC,U87/miR-143,and U87/miR-143 overexpressed with N-RAS. MiR-143 attenuated the accumulation of p65 in nucleus in glioma cells. Overexpression of N-RAS rescued the accumulation of p65 in nucleus inhibited by miR-143. Data represent mean±SD of 3 replicates. *indicate significantly different at *P*<0.05. p65: Red; DAPI: Blue; Merge: Blue+Red.

Constitutive overactivation of NF-κ B signaling is a common event in human cancers and acts as a key factor in cancer development and progression [31-35]. Then, we examined whether miR-143 affects NF-κB pathway. As shown in Figure 4A, the luciferase activity of the NF-κB reporter decreased in miR-143-overexpression cells, suggesting that miR-143 contributes to NF-κB inactivation. MiR-143 overexpression led to reduced phosphorylation of IKKα and elevated IκBα, but the expression of p65, a member of NF-κB family did not changed in U87/miR-143 cells. However, the expression levels of p65 were higher in cytoplasmic extracts of miR-143-overexpressing stable cell lines than in miR-NC cells, while expression of p65 were lower in nuclear extracts; more interestingly, overexpression of N-RAS rescued the accumulation of p65 in nucleus inhibited by miR-143 (Figure 4B). Similarly, we found the expression levels of p65 in miR-143-overexpressing stable cell lines were lower than that of miR-NC group associated with N-RAS group in nucleus by immunofluorescence stain, which indicated that miR-143 may attenuate the accumulation of p65 in nucleus in an N-RAS dependent manner (Figure 4C). Taken together, these results further support the notion that miR-143 functions as a tumor suppressor in glioma and inhibits NF-κB signaling pathway *in vitro* by targeting N-RAS.

### Overexpression of miR-143 suppresses cell proliferation, migration, invasion,and angiogenesis *in vitro*

**Figure5:**
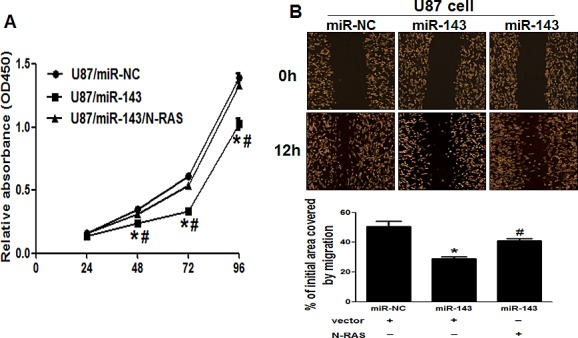

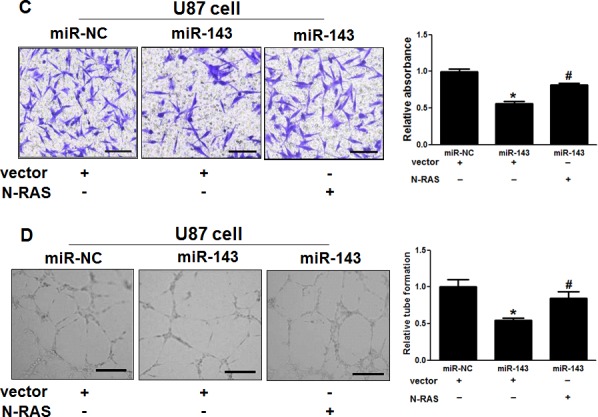
Overexpression of N-RAS reverses the inhibitory effects of miR-143 (A) Overexpression of miR-143 arrested cell proliferation, but this was rescued upon coexpression of exogenous N-RAS in U87 cells. (B) Cells were treated as above. A sterile 200 μl pipette tip was used to scratch the cells to form a wound. The wound gaps were photographed (top) and measured (bottom). Forced expression of miR-143 also markedly reduced the wound-healing rate, and overexpression of N-RAS reverses the inhibitory effects of miR-143. (C) miR-143 overexpression decreased cell invasion in U87 cells. Cells were transfected with miR-143 followed by N-RAS transfection. All cells were subjected to a Matrigel invasion assay. Scale bar, 20 μm. (D)Representative images and quantification of HUVECs cultured on Matrigel-coated plates with conditioned medium from the indicated cells. Scale bar, 20 μm. Data represent mean±SD of 3 replicates. *indicates significant difference compared to control, ^#^indicates significant difference compared to miR-143 treatment alone at *P*<0.05.

Given the important role of N-RAS in regulation of cell proliferation, migration and invasion [36, 37], miR-143-overexpressing U87 cells were used to analyze cell growth and invasion. The results showed that cell growth and migration were attenuated in miR-143-overexpressing U87 cells compared with U87 cells expressing miR-NC (Figure 5A and B). We showed that forced expression of N-RAS restored miR-143-inhibited cell proliferation and migration (Figure 5A and B).

Since invasion and angiogenesis are key characteristics of malignant tumor, we next investigated the effects of miR-143 on invasion and angiogenesis *in vitro*. Restoration of miR-143 dramatically inhibited the normally strong invasive capacity of U87 cells (Figure 5C). Simultaneously, tube formation assay showed that miR-143-transfected group HUVECs presented less tube length, indicating angiogenesis was suppressed (Figure 5D). As expected, forced expression of N-RAS reversed miR-143-mediated suppression of cell invasion and angiogenesis. Thus, our results suggest that overexpression of miR-143 suppresses glioma cell proliferation, migration, invasion and angiogenesis by inhibiting its target N-RAS.

### Overexpression of miR-143 increases chemosensitivity of glioma cells to TMZ by inhibiting its target N-RAS

**Figure6:**
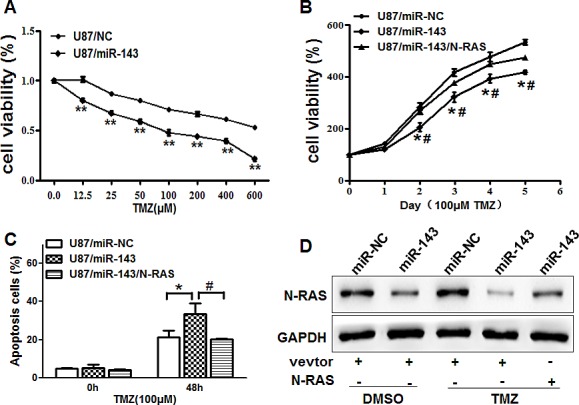
Overexpression of miR-143 increases chemosensitivity of glioma cells to TMZ by inhibiting its target N-RAS (A)U87 cells stably expressing miR-NC or miR-143 were pretreated with various concentration of TMZ for 48 h, and subjected to CCK8 Assay. (B) U87 cells stably expressing miR-NC, miR-143 or miR-143 forced expression of N-RAS were pretreated with 100 μM of TMZ for indicated time points, then subjected to CCK8 Assay, apoptosis analysis by flow cytometry (C) and Western blotting (D). Data represent mean±SD of 3 replicates. *indicates significant difference compared to control, #indicates significant difference compared to miR-143 treatment alone at *P*<0.01.

Resistance to TMZ treatment is one of the major causes for the failure of chemotherapy in treating glioma. Therefore, it is critical to discover new strategies to increase the effectiveness of TMZ for therapeutic purposes. Our results showed that overexpression of miR-143 in U87 cells significantly increased chemosensitivity to treatment of TMZ (Figure 6A). Furthermore, cell growth rate in the presence of TMZ (100μM) was assayed by CCK-8 proliferation assay at different time points, then forced expression of N-RAS reversed miR-143-induced glioma chemosentivity to TMZ (Figure 6B). To further study whether miR-143 and its target N-RAS play a role in cell apoptosis in the presence of TMZ treatment, FACS analysis was performed to detect cell apoptosis rates. The combination of miR-143 and TMZ treatment significantly induced cell apoptosis, whereas forced expression of N-RAS partially abolished the effect induced by miR-143 plus TMZ treatment (Figure 6C). Similarly, we also found that the expression of N-RAS in U87/143 cells treated with TMZ is lower that the non-treated by immunobloting (Figure 6D). These results indicated that miR-143 renders glioma cells more sensitive to TMZ treatment, miR-143 and TMZ combination induced apoptotic effect through targeting N-RAS in glioma cells.

### MiR-143 inhibits tumor growth and angiogenesis *in vivo*

**Figure7:**
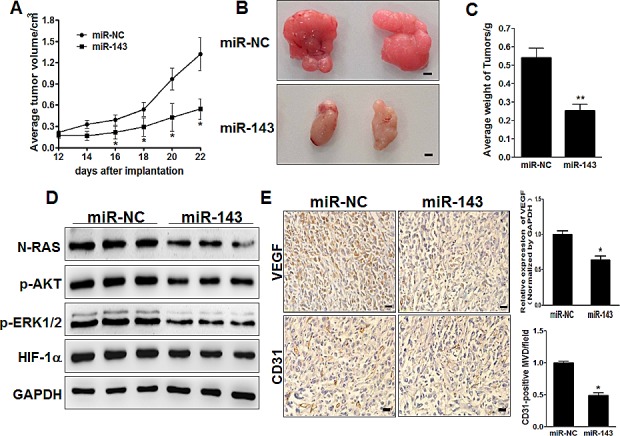
MiR-143 inhibits tumor growth and angiogenesis *in vivo* (A, B,C) Effect of miR-143 on the growth of U87 cells inoculated into nude mice. Male BALB/c nude mice were subcutaneously injected with 5×10^6^ U87 cells infected with lentiviruses harboring miR-NC or miR-143. Tumor volume and weight were monitored over time as indicated, and the tumor was excised and weighed after 24 days. MiR-143 overexpression causes a decrease in tumor volume and weight. Bar=1mm. (D) The expression levels of downstream pathway proteins from the tumor tissues of miR-143 expressing group were lower than that of miR-NC group by immunoblotting assay. (E) The expression levels of VEGF and CD31 were analyzed in tumor tissues by immunohistochemistry with representative images showed. The density of VEGF protein levels was analyzed by qRT-PCR, and CD31 levels were quantified by ImageJ software. Magnification, ×200,Scale bar, 20 μm. Data were presented by mean±SD. *indicated *P<*0.05.

In order to test whether miR-143 attenuates progression of glioma *in vivo*, we engineered U87 cells to stably express miR-NC or miR-143, which were subsequently implanted into both posterior flanks of immunodeficient mice, and tumor sizes were measured after 2 weeks. From the 2^nd^ to 4^th^ week, miR-NC-injected group developed significantly larger tumors than miR-143 group (Figure 7A). MiR-143 stable-expressing cells generated xenografts that were statistically significantly smaller than control (Figure 7B). Meanwhile,the final tumor weight of miR-NC group was much heavier than miR-143 group (Figure 7C). In agreement with *in vitro* studies, the levels of N-RAS from the tumor tissues of miR-143 expressing group were lower than that of miR-NC group by immunoblotting assay (Figure 7D). Moreover, some downstream pathway proteins, such as p-AKT, p-ERK1/2 and HIF-1α were significantly suppressed by miR-143 in glioma tissues (Figure 7D). Consistent with our previous studies, we showed that miR-143 inhibited tumor growth via anti-angiogenesis function. IHC staining revealed that the expression levels of VEGF and CD31 were significantly repressed by miR-143 inglioma tissues (Figure 7E). It was also confirmed that VEGF expression in xenograft tumors were significantly decreased by miR-143. Furthermore, quantitative microvascular density (MVD) analysis showed significant suppression in miR-143 overexpression group, inferring that miR-143 represses angiogenesis in xenografts. Taken together, these results suggest that miR-143 inhibits tumor growth and angiogenesis *in vivo* through targeting N-RAS and other downstream signaling molecules.

## DISCUSSION

MicroRNAs (miRNA), a novel class of regulatory molecules, have been frequently indicated to be dysregulated in diverse human cancers [38]. MiRNAs have been documented to function both as tumor suppressor genes and oncogenes, regulating many cellular events. Recent studies have been reported that miR-143 plays a potential role as a tumor suppressor in many kinds of cancers [12, 13, 36]. However, there are no results referring to the role of miR-143 in glioma at present. In this study, we found that the expression of miR-143 is downregulated in glioma samples compared with normal brain tissues. Moreover, overexpression of miR-143 significantly inhibited glioma cell migration, invasion, and tumor growth.

RAS family play a pivotal role in the transduction of several growth or differentiation factor stimuli [39]. It has been reported that the expression levels of K-RAS and N-RAS are related to the malignant degree of cancers, including glioma, breast cancer, melanoma, and other cancers [40-43]. Recently, accumulating evidence has indicated that expression levels of the RAS family can be regulated by miRNAs. For example, overexpression of miR-145 can inhibits tumor angiogenesis and growth by targeting N-RAS and VEGF. It has been revealed that K-RAS gene is a target of miR-143 in colorectal cancer [17]. In our study, N-RAS oncogene has been experimentally validated as the novel target of miR-143 not only *in vitro*, but also *in vivo*. Firstly, luciferase reporter assay confirmed that miR-143 directly recognize the 3′-UTR of N-RAS transcripts. Secondly, N-RAS expression was significantly abolished in glioma cells which miR-143 stablely-expressed andincreased in glioma cells which miR-143 inhibited. Thirdly, an inverse correlation between N-RAS protein and miR-143 in clinical samples was found. Finally, inhibition of N-RAS expression by miR-143 inhibits constitutive phosphorylation of AKT and ERK1/2. Taken together, the present study provides the first evidence that miR-143 is significant in suppressing glioma cell growth through inhibition of N-RAS translation.

Angiogenesis is a key process in gliomagenesis. AKT and ERK1/2 are upstream regulators of HIF-1α and VEGF, which are the important components for angiogenesis [44, 45]. Consistent with our previous study, phosphorylation of AKT (p-AKT) and ERK1/2 (p-ERK1/2) was significantly inhibited by treatment with miR-143, therefore inhibited the expression of HIF-1α and VEGF; nevertheless, overexpression of N-RAS restored miR-143-inhibited cellular levels of p-AKT,p-ERK1/2, HIF-1α and VEGF. Here, we found that miR-143 functions as an anti-angiogenic regulator in gliomatumorigenesis. Overexpression of miR-143 in glioma cells led to reduced tube formation, number of microvessels *in vivo*, and then impaired tumor growth in tumor xenograft. Interestingly, we also found the interaction between NF-κB signaling pathway and miR-143 in indicated cells by reporter gene assay, immunoblotting and immunofluorescence stain in our study. These results indicate that miR-143 may attenuate the accumulation of p65 in nuclear by targeting N-RAS. Apparently, the precise mechanisms by which miR-143 inactivates NF-κB pathway in glioma need to be further investigated. Taken together, our study highlighted the key role of miR-143 in growth of glioma and suggested that miR-143 could be an ideal target for glioma therapy.

Despite having a better understanding of the molecular events that govern the glioma than ever before, it remains a clinical challenge in the treatment of glioma. Identification of new biomarkers that play a central role in the progression of glioma will benefit diagnosis and targeting therapy of the disease. MiRNAs are differentially expressed in chemosensitive and chemoresistant cells [46, 47]. Several studies have suggested that miRNAs are novel players in the development of chemoresistance. Recent study showed that ectopic miR-34a sensitized the colorectal cancer cells to 5-fluorouracil [48]. MiR-211 in combination with ionizing radiation (IR) and temozolomide significantly induces glioma cells apoptosis and DNA fragmentation [49]. In this study, we found that forced overexpression of miR-143 promoted the effects of TMZ. Flow Cytometer Assay demonstrated that U87 cells with miR-143 stable expression have TMZ higher sensitiveness to apoptosis. Thus, it is important that a miR-143 restoration approach may offer a new modulation strategy to overcome chemoresistance to TMZ treatment in giloma.

In summary, we have identified a link between miR-143 and N-RAS that is a novel constituent of glioma tumorigenesis. Over the past few years, it has been shown that miR-145 and miR-124 target N-RAS in different tumors such as breast cancer, colon cancers and glioblastoma stem cells. We speculate that these tissue-specific miRNAs may contribute to the cognate abnormality via similar pathways. Now despite having a better understanding of the molecular events that govern the glioma than ever before, it remains a clinical challenge in the treatment of glioma. Identification of new biomarkers that play a central role in the progression of glioma will benefit diagnosis and targeting therapy of the disease.

## MATERIALS AND METHODS

### Human tissue samples

Human glioma samples and normal tissues were obtained from the Department of Neurosurgery, the First Affiliated Hospital of Nanjing Medical University. This study was approved by the hospital institutional review board and written informed consent was obtained from all patients. Tissue samples were collected at surgery, immediately frozen in liquid nitrogen and stored until total RNAs or proteins were extracted.

### Cell culture and reagents

Human glioma cell lines, U87 and U251 were maintained in DMEM medium with high glucose and sodium pyruvate, and human embryonic kidney 293T (HEK-293T) cells were cultured in DMEM medium, supplemented with 10% fetal bovine serum and antibiotics (100 units/ml penicillin and 100 mg/ml streptomycin). Cells were incubated at 37°C in a humidified atmosphere of 5% CO_2_ in air. Antibodies against p-AKT (Ser473), AKT, p-ERK1/2 and ERK1/2 were purchased from Cell Signaling Technology (Danvers, MA, USA). Antibodies against HIF-1α,Histone 3 and GAPDH were from Bioworld Technology (Atlanta, Georgia 30305, USA), and antibodies against N-RAS, p65, p-IKKα(Thr23) and IκBα were from Santa Cruz Biotechnology (Santa Cruz, CA, USA), respectively. The growth factor reduced Matrigel were from BD Biosciences (Bedford, MA, USA).

### Lentivirus packaging and stable cell lines

The lentiviral packaging kit was purchased from Open Biosystems (Huntsville, AL, USA). Lentivirus carrying hsa-miR-143 or hsa-miR-negative control (miR-NC) was packaged following the manufacturer's manual. Lentivirus were packaged in HEK-293T cells and collected from the medium supernatant. Stable cell lines were established by infecting lentivirus into U87 and U251 cells and selected by puromycin.

### RNA extraction, reverse transcription PCR and quantitative real time-PCR

RNA was isolated from harvested cells or human tissues with Trizol reagent according to the manufacturer's instruction (Invitrogen, CA, USA). To measure expression levels of miR-143, stem-loop specific primer method was used as described before [50, 51]. Expression of U6 was used as an endogenous control. To determine the mRNA levels of VEGF, total RNAs were reversely transcribed by oligodT primer using PrimeScript RT Reagent Kit (Takara, Dalian, China). Housekeeping gene GAPDH was used as internal control,primers were used as described before [15]. The cDNAs were amplified by qRT-PCR using SYBR Premix DimerEraser (Takara, Dalian, China) on a 7900HT system, and fold changes were calculated by relative quantification (2-^Δ Δ Ct^).

### Immunoblotting

Cells were washed with ice-cold PBS buffer, scraped from the dishes, and centrifuged at 12,000 rpm, 4°C for 15 min. Cell lysates were prepared using RIPA buffer supplemented with protease inhibitors (100 mM Tris, pH 7.4, 150 mMNaCl, 5mM EDTA, 1% Triton X-100, 1% deoxycholate acid, 0.1% SDS, 2mMphenylmethylsulfonyl fluoride, 1mM sodium orthovanadate, 2mM DTT, 2mMleupeptin, 2mMpepstatin). The supernatants were collected and protein concentration was determined using BCA assay (Beyotime Institute of Biotechnology, Jiangsu, China). Tumor tissues from human and nude mice were grinded into powder in liquid nitrogen with RIPA buffer, and the total tissue proteins were extracted as described above. Aliquots of protein lysates were fractionated by SDS-PAGE, transferred to a PVDF membrane (Roche, Switzerland), and subjected to immunoblotting analysis according to the manufacturer's instruction. ECL Detection System (Thermo Scientific, Rockford, IL, USA) was used for signal detection.

### Luciferase reporter assay

The 3′-UTR of N-RAS were synthesized and annealed, then inserted into the SacI and HindIII sites of pMIR-reporter luciferase vector (Ambion) at downstream of the stop codon of the gene for luciferase. For its mutagenesis, the sequences complementary to the binding site of miR-143 in the 3′-UTR (N-RAS:TTCATCTC) was replaced by TACTTGAC. These constructs were validated by sequencing. U87 cells were seeded into a 24-well plate for luciferase assay. After cultured overnight, cells were cotransfected with the wild-type or mutated plasmid, pRL-TK plasmid, and equal amounts of miR-143 or miR-NC. Luciferase assays were performed 24h after transfection using the Dual Luciferase Reporter Assay System (Promega, WI, USA). The primers are as follows: N-RAS forward primer:5′-GCGAGCTCGCTTCTTGTAATTCATCTCTGCTAA-3′, reverse primer:5′-GCAAGCTTTTATGACTAAGCCAAGAACTTCCAG-3′.

To determine the effects of miR-143 on transcriptional activation of VEGF, VEGF reporter plasmid pMAP11-WT or pMAP11-mut [52]was cotransfected into U87 cells with pRL-TK plasmid and equal amounts of miR-143 or miR-NC. Firefly and Renilla luciferase activities were measured 24h later. Experiments were performed in three independent replicates.

### Cell proliferation

To determine the effects of miR-143 on growth of glioma cells, cells were seeded in 96-well plates at confluence of 2000 cells per well. The absorptions of the cells were measured using a CCK8 kit (Dojindo Laboratories, Kumamoto, Japan) according to the manufacturer's instruction at different indicated time points. Data were from three separate experiments with four replications each time.

### Wound healing assay

Cells were cultured until reached 90% confluence in 6-well plates. Cell layers were scratched using a 20μ L tip to form wounded gaps, washed with PBS twice and cultured. The wounded gaps were photographed at different time points and analyzed by measuring the distance of migrating cells from five different areas for each wound.

### Cell invasion assay

Invasion assay was determined using 24-well BD Matrigel invasion chambers (BD Biosciences, Cowley, UK) in accordance with the manufacturer's instructions. 5×10^4^ cells were seeded per well in the upper well of the invasion chamber in DMEM without serum, the lower chamber well contained DMEM supplemented with 10% FBS to stimulate cell invasion. After incubation for 24 h, noninvading cells were removed from the top well with a cotton swab while the bottom cells were fixed with 3% paraformaldehyde, stained with 0.1% crystal violet, and photographed in three independent fields for each well. They were finally extracted with 33% acetic acid and detected quantitatively using a standard microplate reader (at 570 nm). Three independent experiments were conducted in triplicate.

### Tube formation assay

To pretreat the 96-well plate, 50 μL of growth factor-reduced Matrigel thawed on ice was added to each well. The plate was then placed in an incubator to allow the gel to solidify at 37°C for 1 h. Cells were cultured to 90–100% concentration, the old medium was discarded and provided with serum-reduced medium (1% FBS) for 24h. The medium was collected and stored at −80°C. HUVEC cells at subconcentration were switched to EBM-2 basic medium containing 0.2% FBS. In 24 h, the starved HUVEC cells were trypsinized, collected, counted, and resuspended in EBM-2 basic medium. Then cells were mixed with equal volume of the conditioned medium and seeded to Matrigel-pretreated 96-well plate at 10^4^cells/well. In 12h, tube formation was examined under light microscope. The length of the tubes was measured using the Soft Imaging System (Soft Imaging System GmbH, Germany).

### *In Vitro* Chemosensitivity array

Cancer cells were seeded at a density of 4,000 cells per well in a 96-well plate overnight. Freshly prepared TMZ (Sigma-Aldrich, St. Louis, MO, USA) was added with the final concentration ranging from 12.5 to 600 μM. 48h later, cell viability was assayed by CCK8 kit.

### Apoptosis Assay

Apoptosis were measured by flow cytometry as described before[53]. For AnnexinV staining, 5 μL phycoerythrin-Annexin V, 5 μL propidium iodide (BD Pharmingen) and 400μL 1 × binding buffer were added to the samples, which were incubated for 15 min at room temperature in the dark. Then the samples were analyzed by flow cytometry (FACS Canto II, BD Biosciences) within 1 h. The data were analyzed using FlowJo software. Three experiments were performed in triplicate.

### Tumorigenesis in nude mice

Male BALB/c nude mice (6-weeks-old) were purchased from Shanghai Laboratory Animal Center (Chinese Academy of Sciences, Shanghai, China) and maintained in special pathogen-free (SPF) condition for one week. Animal handling and experimental procedures were in accordance with the Guide for the Care and Use of Laboratory Animals, and approved by the Animal Experimental Ethics Committee of Nanjing Medical University. U87 cells stably expressing miR-143 or miR-NC were injected subcutaneously into both flanks of nude mice (5×10^6^ cells in 100 μl). Tumor sizes were measured using vernier caliper every two days when the tumors were apparently seen and tumor volume was calculated according to the formula: volume = 0.5×Length×Width^2^. 24 days after implantation, mice were sacrificed and tumors were dissected. Total proteins and RNAs were extracted for immunoblotting and qRT-PCR. Tumors were formalin-fixed, paraffin-embedded, and sectioned at 5μm for VEGF (Santa Cruz, CA, USA) and CD31 (Abcam, Cambridge, UK) immunohistochemical staining under the standard procedure as described before [54].

### Statistical analysis

All experiments were performed three times and data were analyzed with GraphPad Prism 5 (La Jolla, CA, USA). The correlation between miR-143 expression and N-RAS levels in glioma tissues were analyzed using Spearman's rank test. Statistical evaluation for data analysis was determined by *t*-test. The differences were considered to be statistically significant at *P*< 0.05.
